# Unveiling the anticancer potential of the ethanolic extract from *Trichoderma asperelloides*

**DOI:** 10.3389/fphar.2024.1398135

**Published:** 2024-05-01

**Authors:** Ana Carolina R. Oliveira, Flávia Santiago De Oliveira, Ana Flávia Bráz, Jamil S. Oliveira, Jane Lima-Santos, Adriana A. M. Dias

**Affiliations:** ^1^ Laboratory of Inflammation and Cancer, Department of Genetics, Ecology and Evolution, Institute of Science Biological, Universidade Federal de Minas Gerais, Belo Horizonte, MG, Brazil; ^2^ Physical Chemistry of Proteins and Enzymology, Institute of Biological Sciences, Universidade Federal de Minas Gerais, Belo Horizonte, MG, Brazil; ^3^ Laboratory of Immunobiology, Department of Biological Sciences, Universidade Estadual de Santa Cruz, Ilhéus, BA, Brazil

**Keywords:** *Trichoderma asperelloides*, drug discovery, anticancer, cytotoxicity, synergism, cancer, *Trichoderma*, antineoplastic action

## Abstract

The discovery of new therapeutic alternatives for cancer treatment is essential for improving efficacy and specificity, overcoming resistance, and enabling a more personalized approach for each patient. We investigated the antitumor activity of the crude ethanolic extract of the fungus *Trichoderma asperelloides* (ExtTa) and its interaction with chemotherapeutic drugs. It was observed, by MTT cytotoxicity assay, that ExtTa significantly reduced cell viability in breast adenocarcinoma, glioblastoma, lung carcinoma, melanoma, colorectal carcinoma, and sarcomas cell lines. The highest efficacy and selectivity of ExtTa were found against glioblastoma T98G and colorectal HCT116 cell lines. ExtTa is approximately four times more cytotoxic to those tumor cells than to non-cancer cell lines. A synergistic effect between ExtTa and doxorubicin was found in the treatment of osteosarcoma Saos-2 cells, as well as with 5-fluorouracil in the treatment of HCT116 colorectal carcinoma cells using CompuSyn software. Our data unravel the presence of bioactive compounds with cytotoxic effects against cancer cells present in *T. asperelloides* ethanolic crude extract, with the potential for developing novel anticancer agents.

## Introduction

Cancer encompasses a wide diversity of diseases with different response profiles to therapies (Hanahan, 2022). Despite recent therapeutic advances and the numerous approaches available in the clinic today, lack of response to treatments, development of resistance, relapses, and adverse effects are often observed (Dagogo-Jack and Shaw, 2018; Siegel et al., 2021). Although chemotherapy is widely used, adverse effects such as hematological changes, and toxicity to the kidneys, liver, heart, nervous system, among others are often associated with the treatment (Meredith and Dass, 2016; Dagogo-Jack and Shaw, 2018). Additionally, the emergence of antineoplastic-resistant cancer cells and tumor recurrence represent the main causes of treatment failure, mainly in metastatic cancer (Siegel et al., 2021).

Precision medicine, aiming to provide personalized cancer treatment and improve effectiveness, remains one of the healthcare system’s biggest challenges. Solutions include the discovery of novel drugs as well as the identification of possible drug synergies (Lawrence et al., 2014a; Zagidullin et al., 2019a).

Fungi are a well-known and valuable natural source of bioactive compounds of therapeutic relevance (Newman and Cragg, 2015), and species of *Trichoderma* have garnered particular attention (Deng et al., 2013; Li et al., 2015; Gao et al., 2021; Zhang et al., 2021). Secondary metabolites with microbicidal, immunomodulatory, and antitumor activity have been isolated from the extract of many species of *Trichoderma* species, including paracelsin, L-lysine α-oxidase, and Trichodermin (Schmoll and Schuster, 2010; Pokrovsky et al., 2013; Gao et al., 2021; Lukasheva et al., 2021).

*Trichoderma asperelloides* hold economic importance in agriculture as a biofungicide agent and for promoting the radicular growth of plants (Guo et al., 2023; Woo et al., 2023). Previous research by Santos SS et al. described antibacterial properties against *Staphylococcus aureus* (Santos et al., 2018) and leishmanicidal properties against the promastigote forms of *Leishmania amazonensis* attributed to the ethanolic extract of *T. asperelloides* (Lopes et al., 2020).

In this study, we investigated the antiproliferative potential of the ethanolic extract of *T. asperelloides* (ExtTa) in various cancer cell lines. Remarkably, our study compared the biological effect of ExtTa with the cytotoxicity of doxorubicin (DOX), a potent chemotherapeutic commonly used in the clinic to treat a wide range of cancers including carcinomas, sarcomas, and hematological cancers. We also analyzed the effect of combining ExtTa with DOX and 5-Fluorouracil (5-FU), a chemotherapeutic commonly used in the treatment of digestive system cancers, especially colorectal cancer. To our knowledge, this research represents the pioneering exploration of *T. asperelloides* anticancer properties, shedding light on its unexplored therapeutic potential in this context.

## Materials and methods

### *Trichoderma asperelloides* culture and ethanolic extract preparation

The *T. asperelloides* LIBASP02 strain (Lopes et al., 2020) was cultured on Potato Dextrose Agar (PDA, HiMedia) in Petri dishes at 28°C for 7 days. The crude ethanolic extract of the mycelium and spores was obtained by washing the cultures with 95% ethanol (Merck) (Fukuzawa et al., 2008). The ethanolic suspension was incubated under agitation in an orbital shaker (Gerhardt) at 100 rpm for 24 h and then centrifuged at 10,000 × g for 20 min (BR4i-Jouan centrifuge). The final crude ethanolic extract (ExtTa) was lyophilized and solubilized in 100% DMSO (Sigma), resulting in a final concentration of 197 mg/mL.

### Cell lines and culture

Human cancer cell lines T98G (glioblastoma), MDA-MB-231 (breast adenocarcinoma), A549 (lung carcinoma), HT1080 (fibrosarcoma), LB373 (melanoma), Saos-2 (osteosarcoma), HCT116 (colorectal carcinoma) and WiDr (colorectal adenocarcinoma) were used. The human non-cancer cell lines GM637, HEK-293T, and the non-cancer mice cells NIH/3T3 and L929 were used as references. L929 and GM637 cells were kindly provided by Dr. Luiz Fernando Lima Reis (Hospital Sírio Libanês - São Paulo, Brazil), and all other cell lines are from ATCC. All cell lines were kept cryopreserved in liquid-phase nitrogen in the Laboratory of Inflammation and Cancer (LINC) at the Universidade Federal de Minas Gerais (UFMG—Minas Gerais, Brazil). All cell lines were kept cryopreserved in liquid-phase nitrogen in the Laboratory of Inflammation and Cancer (LINC) at the Universidade Federal de Minas Gerais (UFMG). Cell cultures were maintained in a humidified atmosphere regulated at 5% CO2 and 37°C in the specific medium for each cell line: Dulbeccos’s Modified Eagle’s Medium (DMEM - Gibco) for lines HEK-293T, NIH/3T3, L929, HCT116, A549, HT1080, DMEM supplemented with non-essential amino acids (Sigma) for lines GM637, Saos-2, T98G, WiDr; Iscove’s Modified Dulbecco’s Medium (Gibco) for LB373; and RPMI-1640 Medium (Gibco) for MDA-MB-231—supplemented with 10% FBS (Sigma), 2 mM L-glutamine (Sigma), 10 μg/mL penicillin-streptomycin (Sigma). Subculture was performed using trypsin 0.25%—EDTA 0.53 mM (Gibco) for cell detachment. All lines tested negative for *mycoplasma* contamination in a PCR assay using primers as described by Young et al (Young et al., 2010).

### Cell viability assay

The MTT (3-(4,5-dimethylthiazol-2-yl)-2,5-diphenyltetrazolium bromide, Sigma Aldrich, St. Louis, MO, United States) assay was used to assess cell viability in response to each treatment (van Meerloo et al., 2011; Stockert et al., 2018). Briefly, cells were cultured in wells of 96-well plates for 48 h in the presence of varying concentrations of ExtTa, DOX, or 5-FU. The number of cells seeded per well was determined in a pilot assay for standardization. Cells were seeded at concentrations ranging from 0.5 to 2.0 × 10⁵ cells/well, depending on the cell type and its growth rate. 0.5% DMSO was added to all wells, including those containing control cells and those with treated cells, to eliminate any potential bias caused by the vehicle concentration present in samples treated with higher concentrations of ExtTa and DOX. DMSO 0.5% was used as survival control and DMSO 20% as a 100% cell death control. Subsequently, MTT solution (5 mg/mL) was added (0.5 mg/mL final concentration) to each well, and cells were incubated for 3 h. Then, cell media was removed, and a 1:1 v/v N'N'Dimethylformamide solution (Synth) in 10% SDS (Affymetrix) was added for formazan solubilization. Absorbance was recorded at 595 nm using a Multiskan Spectrum reader (Thermo Scientific). Cell viability was quantified using the following equation: $\%\;Cell\;viability=\frac{Absorbance\;individual\;test\;group-absorbance\;blank\;group\;}{Absorbance\;control\;group-absorbance\;blank\;group}$ . The half-maximal inhibitory concentration (IC_50_) was calculated based on the dose-response inhibition curve, using the GraphPad Prism software (v. 8.0.1, San Diego, California, United States). All assays were performed in technical quadruplicates with three independent biological replicates.

### Determination of the selectivity index

Non-cancer cell lines GM637, HEK-293T, NIH/3T3 and L929 were used as reference for selectivity index (SI) calculation following the equation (López-Lázaro, 2015): $SI=\frac{IC50\;non-cancer\;cell\;line}{IC50\;cancer\;cell\;line}$

### CompuSyn analysis

The CompuSyn software (CompuSyn, Inc., Paramus, NJ, United States) calculates a Combination Index (CI) that assigns numerical values to levels of synergism, additivity, or antagonism between compounds. The combinatorial effect of 8 different dose combinations of the ExtTa, DOX or 5-FU (dilutions 1:2, in a constant ratio) was calculated according to the Chou-Talalay Combination Index method (Chou, 2006; Chou, 2010) based on the cell viability assessed by MTT assay. The fraction of inhibited cells (Fa) in each dose of the treatment or the combination was calculated. Fa = 0 means non-inhibitory effect and Fa = 1 means a 100% inhibitory effect. The half-maximal inhibitory concentration (IC_50_) of the drug is calculated by the dose-effect curve and median-effect. The x-axis intercept (logD) represents the medium-effect dose of IC_50_. The CI was automatically determined by the CompuSyn software based on the median effect equation. CI < 1 points to synergism; CI = 1 means additive effect, and when CI > 1 the interaction is considered antagonistic. Additionally, the dose-reduction index (DRI) was calculated to assess if it is possible to reduce the dosage of the compounds to achieve the same inhibitory effect in a combination scenario. DRI >1 represents the possibility of a favorable dose reduction; DRI = no dose-reduction index; DRI <1 not favorable dose-reduction (Chou, 2010).

### Statistical analysis

In the cell viability assays, non-linear regression was applied to find the IC_50_. Results are presented as the mean ± SD of three independent experiments in triplicates. Statistical analyses and graphs were performed using the GraphPad Prism Version 8 program.

## Results

### The ethanolic extract of *T. asperelloides* impairs cancer cells proliferation *in vitro*

**FIGURE 1:**
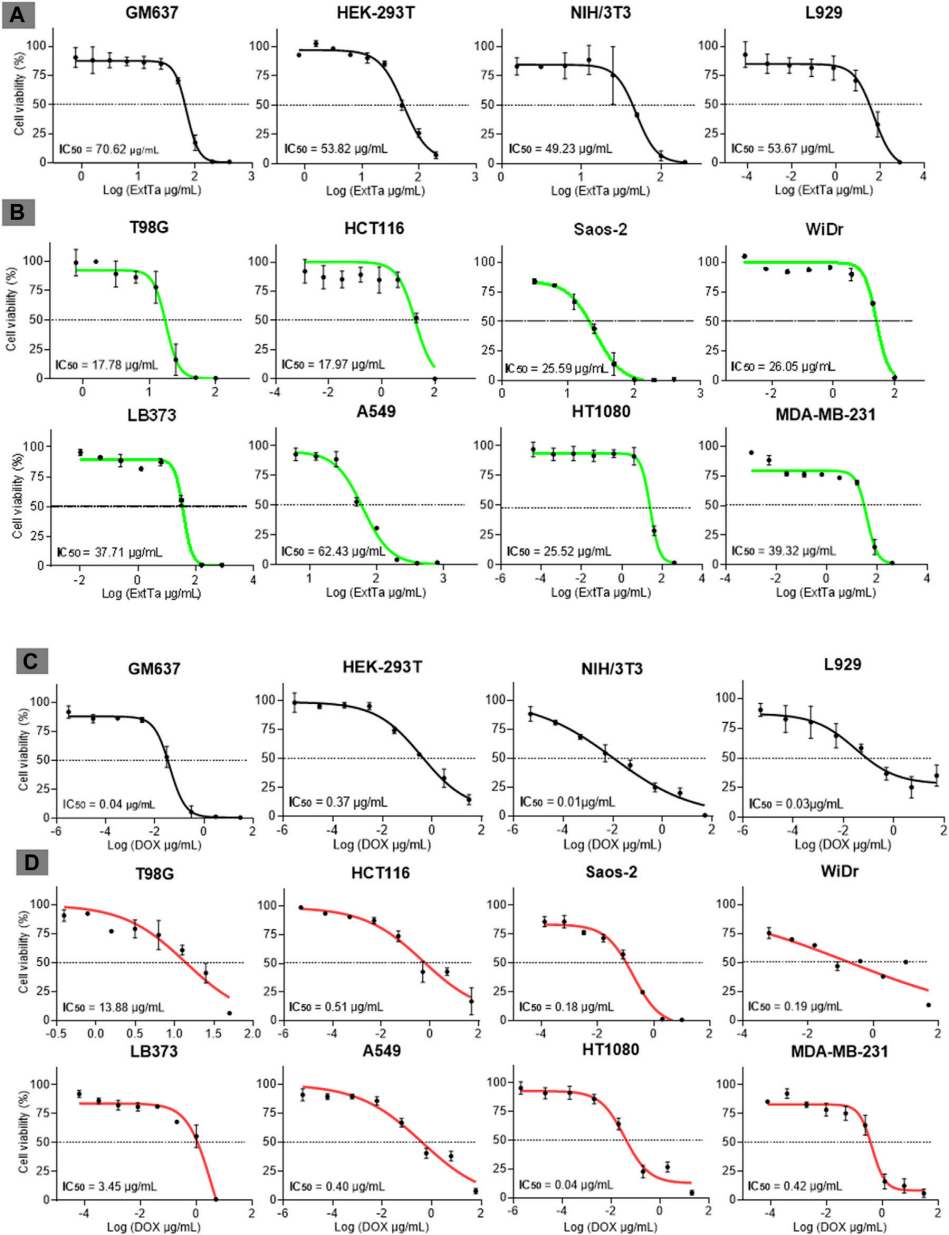
Dose-response curves of ExtTa and DOX. Dose-response curves of ExtTa (ethanolic extract from *Trichoderma asperelloides*) for non-cancer cell lines GM637, HEK-293T, mice NIH/3T3, and L929 **(A)**. Dose-response curves of ExtTa for tumor cell lines T98G, HCT116, Saos-2, WiDr, LB373, A549, HT1080, and MDA-MB-231 **(B)**. Dose-response curves of DOX (doxorubicin) for non-cancer human cell lines GM637, HEK-293T, murine NIH/3T3, and L929 **(C)**. Dose-response curves for DOX for tumor cell lines T98G, HCT116, Saos-2, WiDr LB373, A549, HT1080, MDA-MB-231 **(D)**. Values shown are the means of three independent experiments (*n* = 3). Dose-response curves were generated using GraphPad Prisma Version 8.0.1 software, employing a non-linear regression analysis model to obtain IC_50_ (half-maximal inhibitory concentration of a drug) values.

ExtTa exhibited cytotoxicity towards cells in a dose-dependent manner. The mean IC_50_ values for non-cancer lines GM637, HEK-293T, NIH/3T3, and L929 after 48 h post-treatment with ExtTa were 70.62 ± 5.15, 53.82 ± 11.07, 49.23 ± 7.83, and 53.67 ± 6.54 μg/mL, respectively (Figure 1A). Most tumoral cell lines demonstrated greater sensitivity to ExtTa cytotoxic activity compared to non-cancer cells: T98G (mean IC_50_ = 17.78 ± 3.20 μg/mL), MDA-MB-231 (IC_50_ = 39.32 ± 9.87 μg/mL), A549 (IC_50_ = 53.67 ± 0.08 μg/mL), HCT116 (IC_50_ = 17.97 ± 4.34 μg/mL), LB373 (IC_50_ = 37, 71 ± 2.06 μg/mL), HT1080 (IC_50_ = 25.52 ± 6.66 μg/mL), WiDr (IC_50_ = 26.05 ± 6.08 μg/mL) and Saos-2 (IC_50_ = 25.59 ± 2.76 μg/mL) (Figure 1B). Treatment with ExtTa resulted in a dose-dependent decrease in cell proliferation across different levels. Notably, T98G (glioblastoma) and HCT116 (colorectal carcinoma) cells exhibited the highest sensitivity to the cytotoxic effects of ExtTa.

All non-tumoral cell lines tested in our study, GM637 (IC_50_ = 0.04 ± 0.03 μg/mL), HEK-293T (IC_50_ = 0.37 ± 0.07 μg/mL), NIH/3T3 (IC_50_ = 0.01 ± 0.01 μg/mL), and L929 (IC_50_ = 0.03 ± 0.08 μg/mL), exhibited sensitivity to DOX (Figure 1C). Among the tumor cell lines, varying profiles of cytotoxicity to DOX treatment were observed: T98G (IC_50_ = 13.88 ± 2.91 μg/mL), MDA-MB-231 (IC_50_ = 0.42 ± 0.09 μg/mL), A549 (IC_50_ = 1.39 ± 0.08 μg/mL), HCT116 (IC_50_ = 0.51 ± 0.05 μg/mL), LB373 (IC_50_ = 3.45 ± 2.01 μg/mL), HT1080 (IC_50_ = 0.04 ± 0.01 μg/mL), WiDr (IC_50_ = 0.19 ± 0.14 μg/mL) and Saos-2 (IC_50_ = 0.18 ± 0.14 μg/mL) (Figure 1D). Notably, non-cancer lines GM637, NIH/3T3, and L929 as well as the tumor cell line HT1080, exhibited higher sensitivity to DOX. Conversely, T98G and LB373 cell lines demonstrated less sensitivity to the cytotoxic effects of this drug.

### The ExtTa demonstrated selectivity for cancer cell lines

The selectivity indices for ExtTa and DOX are presented in Table 1. The cytotoxicity of ExtTa was approximately 2 times higher for Saos-2, MDA-MB-231, LB373, and HT1080 cell lines, and 3 to 4 times higher for T98G and HCT116 cells compared to non-cancer cell lines. The A549 cell line exhibited less sensitivity to ExtTa cytotoxicity, and no dose-dependent selectivity was observed for this tumor cell (Table 1).

**TABLE 1:** ExtTa and DOX Selectivity Index (SI) values for tumor cell lines using GM637, HEK-293T, NIH/3T3, and L929 non-cancer cells as reference.

Selectivity Index (SI*)								
Reference non tumor cells	GM637		HEK-293T		NIH/3T3		L929	
Cancer cell lines	Treatment							
	ExtTa	DOX	ExtTa	DOX	ExtTa	DOX	ExtTa	DOX
**T98G**	**3.97**	NS	**3.03**	NS	**2.68**	NS	**3.13**	NS
**MDA-MB-231**	1.80	NS	1.37	NS	1.21	NS	1.42	NS
**A549**	1.13	NS	0.86	NS	0.76	NS	0.89	NS
**HCT116**	**3.93**	NS	**2.99**	NS	**2.65**	NS	**3.10**	NS
**LB373**	1.87	NS	1.43	NS	1.26	NS	1.48	NS
**HT1080**	**2.77**	1.00	**2.11**	9.25	**1.87**	NS	**2.18**	NS
**WiDr**	**2.71**	NS	**2.07**	1.95	**1.83**	NS	**2.14**	NS
**Saos-2**	**2.76**	NS	**2.10**	2.05	**1.86**	NS	**2.17**	NS

*SI, ratio between the IC50 of non-cancer cells and cancer cells. The values in bold represent the highest selectivity indices (SI)

NS = SI < 1.0.

In our study, DOX exhibited selectivity only for the HT1080 cell line compared to the non-cancer cell line HEK-293T, being 9.25 times more cytotoxic for this tumor cell line than for non-cancer cell (Table 1).

The results indicated that the selectivity index (SI) of ExtTa surpassed that of DOX for most of the evaluated cells. ExtTa demonstrated higher cytotoxicity for the cancer cell lines HCT116 and T98G than for the non-cancer cell line GM637, highlighting the selectivity of the ExtTa and its minor impact on healthy cells, a characteristic not observed in DOX-treated cells.

### ExtTa has a synergistic effect with DOX and 5-FU chemotherapeutics

**FIGURE 2:**
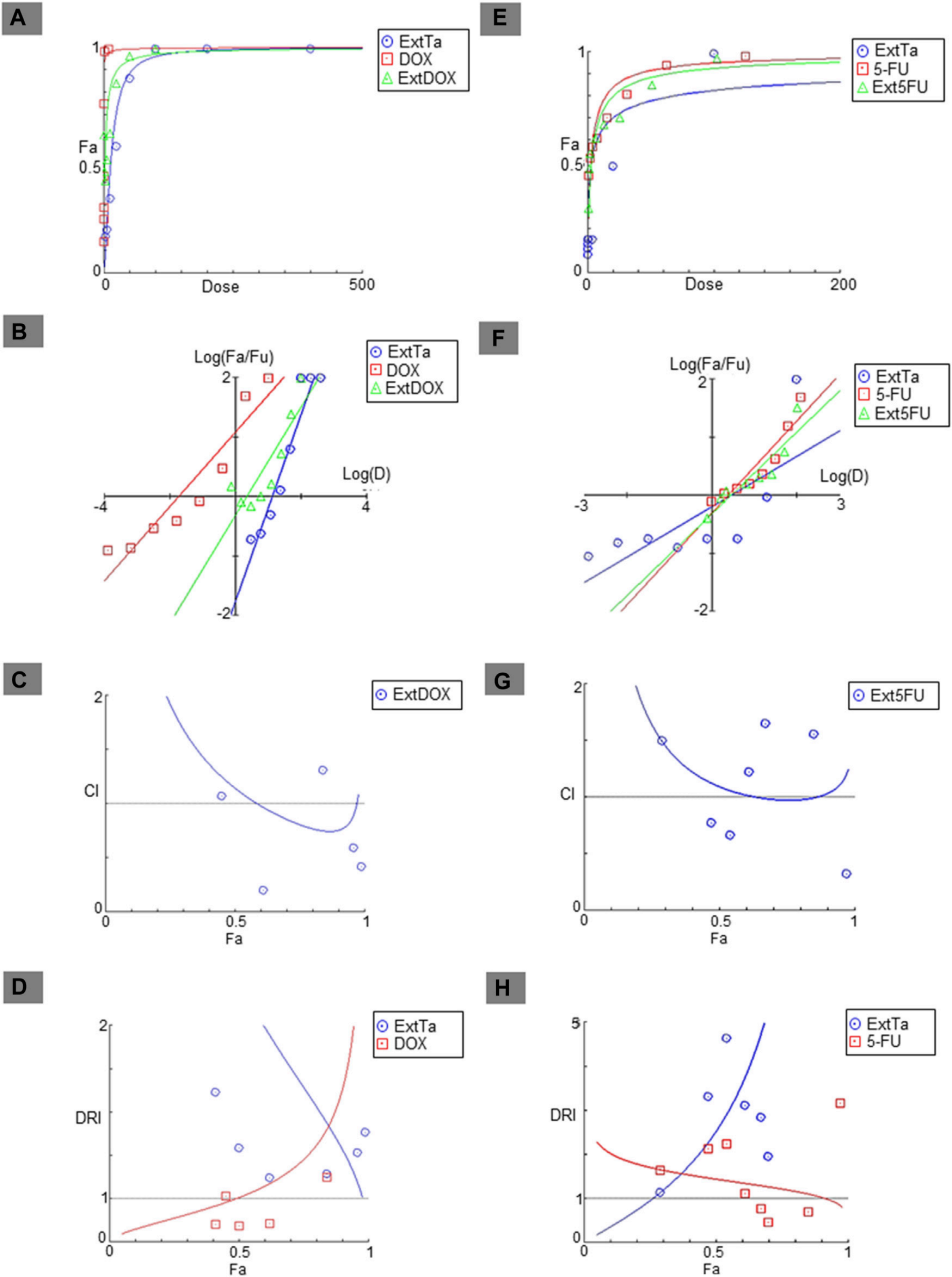
Fa-CI plot of combined treatments of ExtTa with DOX in Saos-2 osteosarcoma cells and with 5-FU in HCT116 colorectal cancer cells. The combination index was calculated using CompuSyn software. Dose-effect curve for ExtTa + DOX **(A)** and ExtTa + 5-FU **(E)**; Median-effect plot for ExtTa + DOX **(B)** and ExtTa + 5-FU **(F)**. Combination index (CI) for ExtTa + DOX **(C)** and ExtTa + 5-FU **(G)**; Dose-reduction index (DRI) for the combination of the ExtTa + DOX **(D)** and ExtTa + 5-FU **(H)**. Fa, fraction affected; ExtTa, Ethanolic Extract *T. asperelloides*; DOX, doxorubicin; ExtDOX, combined treatments of Ethanolic Extract *T. asperelloides* and doxorubicin; 5-FU, 5-fluorouracil; Ext5FU, combined treatments of Ethanolic Extract *T. asperelloides* and 5-fluorouracil.

Addressing the challenge of making cancer treatment more personalized and effective remains one of the major hurdles in the healthcare system. Combinations of multidirectional synergistic drugs may offer effective and sustained clinical responses (Lawrence et al., 2014b; Zagidullin et al., 2019b). With this aim, the effects of combining ExtTa with DOX were evaluated in the tumor cell lines MDA-MB-231, Saos-2, and HT1080, which are sensitive to ExtTa and where DOX is commonly used for clinical treatment of these tumor types. Additionally, the combination of ExtTa with 5-Fluorouracil (5-FU) was assessed in the colorectal carcinoma cell line HCT116, considering that this chemotherapeutic agent is used as the first-line treatment for this type of tumor. The combinatorial effect of ExtTa and DOX on the Saos-2 osteosarcoma cell line was graphically represented in Figure 2 through dose-effect curves (Figure 2A), median-effect (Figure 2B), CI graphs (Figure 2C), and dose-reduction index (Figure 2D). The co-treatment of ExtTa with DOX was found to be more cytotoxic than each isolated treatment (Figure 2; Table 2). The most significant synergistic effects were observed at the lowest and highest concentrations of the compounds (Figure 2C; Table 2). Favorable ExtTa dose reduction was evident across all the inhibitory fractions (Fa) evaluated. Favorable DOX dose reduction was observed on the inhibition fractions from 0.45 to 0.97 (Figure 2D; Supplementary Table S1). The effect of ExtTa and 5-FU on the HCT116 colorectal carcinoma cell line was graphically represented in Figure 2 through dose-effect curves (Figure 2E), median-effect (Figure 2F), CI graphs (Figure 2G), and dose-reduction index (Figure 2H).

**TABLE 2:** Evaluation of the combinatorial effect of ExtTa with DOX and 5-FU in Saos-2 osteosarcoma and HCT116 colorectal cancer cells, respectively.

	ExtTa (μg/mL)	DOX (μg/mL)	Fa	CI	Effect
Saos-2	0.781	0.006	0.61	0.20203	Synergism
	1.563	0.112	0.45	1.06995	Antagonism
	3.125	0.224	0.41	2.75892	Antagonism
	6.250	0.450	0.50	3.14533	Antagonism
	12.500	0.900	0.62	3.04077	Antagonism
	25.000	0.180	0.84	1.31011	Antagonism
	50.000	0.360	0.96	0.59225	Synergism
	100.000	0.720	0.99	0.41492	Synergism

	ExtTa (μg/mL)	5-FU (μg/mL)	Fa	CI	Effect
HCT116	0.32	0.49	0.29	1.49333	Antagonism
	0.63	0.98	0.47	0.77004	Synergism
	1.25	1.95	0.54	0.66478	Synergism
	2.50	3.90	0.61	1.22185	Antagonism
	5.00	7.81	0.67	1.64750	Antagonism
	10.00	15.63	0.70	2.68580	Antagonism
	20.00	31.25	0.85	1.55373	Antagonism
	40.00	62.50	0.97	0.32131	Synergism

ExtTa, *T. asperelloides* ethanolic extract; DOX, doxorubicin; 5-FU, 5-Fluorouracil; Fa, fraction inhibition; CI, combination index.

For colorectal carcinoma HCT116 cells, we also observed a synergistic action of the ExtTa and 5-FU (Figure 2G). The combination index indicated synergistic action for specific dose combinations (Table 2). For the synergistic combination of ExtTa with 5-FU, DRI values demonstrated dose reduction for both compounds for Fa from 0.3 to 0.90 (Figure 2H). As a result, the IC_50_ values of each drug in combination decreased 2.53 and 1.44 times for ExtTa and 5-FU, respectively (Supplementary Table S2).

For the MDA-MB-231 breast cancer cell line, synergism between ExtTa and DOX was observed only at high doses, without an indication of a favorable dose reduction (Supplementary Figure S1).

An antagonistic effect was found when ExtTa and DOX were used simultaneously in the treatment of the HT1080 fibrosarcoma cell line (Supplementary Figure S2). In this case, the combination was less effective in inducing cytotoxicity than each compound isolated in all tested doses.

## Discussion

Cancer is a worldwide health problem, and despite the availability of more than a hundred chemotherapeutics, new drugs are still necessary to improve efficacy, circumvent undesirable adverse effects, and impair resistance and cancer recurrence. Precision medicine calls for new therapeutic drugs to provide personalized treatment and improve patient’s life quality. In this study, we sought to investigate the existence of active compounds with antitumor activity in the ethanolic extract of the fungus *T. asperelloides*. ExtTa cytotoxicity was assessed in a panel of tumoral cells and compared with the effect of the drug DOX already used in clinics. Besides having a greater selectivity for all tumor cell lines tested than DOX, ExtTa also presented a synergistic action with DOX and with 5-FU in some tumor types. A reduction in the favorable dose of the drugs was also observed in some cases.

ExtTa exhibited cytotoxic activity against glioblastoma, a highly aggressive type of cancer characterized by cells capable of infiltrating the entire brain (Bryukhovetskiy et al., 2018; Goldsmith, 2019). Despite current treatments involving primary tumor surgical removal followed by post-surgical radiation and subsequent chemotherapy administration, the results remain generally unsatisfactory (Bryukhovetskiy et al., 2018; Zagidullin et al., 2019b), and the development of new therapeutic modalities is mandatory (Lawrence et al., 2014b). Our *in vitro* data indicate that it is worth evaluating the effect of the ExtTa on the development of glioblastoma tumors *in vivo.*

Another promising finding in this study was the selective cytotoxic action of ExtTa against human colorectal carcinoma HCT116 cells. Colorectal carcinoma is one of the most frequent malignant tumors in humans and has a high risk of death worldwide. This type of cancer represents 10.2% of all new cancer diagnoses and 9.2% of mortality, being the second cause of death by cancer among women, with an average survival rate of 55% (Bray et al., 2018; Siegel et al., 2021). Currently, there are several types of colorectal cancer therapy, such as the chemotherapeutic drug 5-FU combined with irinotecan (Van Cutsem et al., 2012; Davis et al., 2022). However, even with significant advances, available therapies often lead to multiple chemoresistance, and relapses, or are only palliative in many patients (Martín et al., 2021). Therefore, new drugs or combinations are urgently needed to increase the effectiveness of chemotherapeutic agents, prevent progression, and reduce mortality from this disease.

The selectivity index (SI) is a widely used parameter to assess whether there is a difference in the harmfulness of a compound for non-cancer and cancer cells in *in vitro* cytotoxicity assays. It expresses how much more cytotoxic the tested compound is for tumor cells compared to non-cancer cells (Indrayanto et al., 2021; Romero-Arguelles et al., 2022). It is known that one of the biggest challenges of antitumor drugs is the ability to be selective for tumor cells without causing harm to healthy cells (Jamalzadeh et al., 2017). The non-cancer cell lines used in this study are often used for drug screenings (Schwarz et al., 2008; Hu et al., 2018; Cannella et al., 2020). Our data show that the ExtTa was more selective for tumor cells in general than DOX. It is worth exploring this feature in further studies since one of the expected characteristics of a new antitumor agent is its ability to eliminate tumor cells while being less aggressive for normal cells.

Although DOX has good efficacy in the treatment of different types of solid tumors, due to its lack of selectivity, DOX presents many undesirable adverse effects such as stomatitis, gastrointestinal disturbances, nausea, fatigue, alopecia, and cardiotoxicity, which is a major concern during therapy (Carvalho et al., 2009; Meredith and Dass, 2016; Mollaei et al., 2021). The combination of DOX with drugs that can have synergistic effects can circumvent some of the undesirable effects by promoting a reduction of DOX dosage, consequently minimizing toxicity and improving the therapeutic efficacy by avoiding drug resistance resulting from tumor heterogeneity (Lawrence et al., 2014a; Gottesman et al., 2016; Zagidullin et al., 2019a). The combination index resultant from the simultaneous treatment with ExtTa and DOX pointed out a synergistic cytotoxic action on the osteosarcoma line, which suggests that this combination would allow an increase in the chemotherapeutic effect of DOX. The results also showed a favorable dose reduction (DRI>1) as shown in FIGURE 2D, so it is possible to reduce the dose of DOX by up to 6.63 times while maintaining its effectiveness when this drug is associated with ExtTa (Supplementary Table S1). Thus, our data point to potential use for ExtTa or its isolated components as adjuvants in DOX-based osteosarcoma chemotherapy. ExtTa in combination with DOX could result in a more selective and effective therapy with a reduction of adverse undesirable effects and tumor recurrence (Chou, 2006; Kazantseva et al., 2022).

Synergism was also observed when treating the colorectal cancer cell line HCT116 with the ExtTa combined with 5-FU, as well as a favorable reduction in the dose of each compound. By increasing the therapeutic efficacy of conventional agents in combination, it is possible to obtain positive results such as maintaining or increasing the same efficacy without causing toxicity due to the reduction of the dose. These results confirm that a therapeutic approach in combination with ExtTa or its isolated components represent a potential strategy to reduce the therapeutic dosage of chemotherapeutic agents, increase the effect, and minimize unwanted drug reactions.

Although the composition of ExtTa and its bioactive compounds have not yet been identified and isolated, our findings demonstrate that ExtTa exhibits selective anticancer activity, at least against cell lines derived from glioblastoma (T98G) and colorectal carcinoma (HCT116). Additionally, ExtTa exhibited synergistic anticancer effects with the chemotherapeutics DOX and 5-FU on osteosarcoma cells (Saos-2) and colorectal carcinoma (HCT116), respectively.

In conclusion, the ExtTa may represent an important potential source for the discovery of novel molecules with anticancer activity, thereby increasing the range of treatment options and drug combinations aimed at reducing doses, undesirable adverse symptoms, and improving efficacy. Ongoing studies conducted by our research group aim to identify, isolate, and characterize the potential active substances present in ExtTa, as well as to investigate the underlying mechanisms of its action.

## Data Availability

The original contributions presented in the study are included in the article/Supplementary Material, further inquiries can be directed to the corresponding author.

## Supplementary material

SUPPLEMENTARY FIGURE S1 Fa-CI plot of combined treatments of ExtTa and DOX in MDA-MB-231 adenocarcinoma breast cells. The combination index was calculated using CompuSyn software. Dose-Effect Curve **(A)**; Median-effect plot **(B)**. Combination index **(C)**; Dose-reduction index (DRI) **(D)**. Fa: fraction affected; ExtTa: Ethanolic Extract T. asperelloides; DOX: doxorubicin; ExtDOX: combined treatments of Ethanolic Extract T. asperelloides and doxorubicin.

SUPPLEMENTARY FIGURE S2 Fa-CI plot of combined treatments of ExtTa and DOX in HT1080 fibrosarcoma cells. The combination index was calculated using CompuSyn software. Dose-Effect Curve **(A)**; Median-effect plot **(B)**. Combination index (CI) **(C)**; Dose-reduction index (DRI) **(D)**. Fa: fraction affected; ExtTa: Ethanolic Extract T. asperelloides; DOX: doxorubicin; ExtDOX: combined treatments of Ethanolic Extract T. asperelloides and doxorubicin.

## Abbreviations

ExtTa, T. asperelloides ethanolic extract; DOX, doxorubicin; 5-FU, 5-Fluorouracil; Fa, fraction inhibition; CI, Combination index.
