# Supplementary material for: Unveiling the anticancer potential of the ethanolic extract from Trichoderma asperelloides
Source: Front Pharmacol. 2024 May 1;15:1398135. doi: 10.3389/fphar.2024.1398135 (PMC11094271; doi:10.3389/fphar.2024.1398135)
Supplement: Supplementary file 1 [file Table1.docx]

**SUPPLEMENTARY TABLE 1 - Dose reduction index (DRI) for ExtTa and DOX for a given inhibition effect in Saos-2 cell line**

*Values calculated at experimental points. DRI: dose reduction index, ExtTa: *T. asperelloides* ethanolic extract; DOX: doxorubicin; Fa: fractional inhibition.

| Fa | µg/mL | | DRI* | |
| --- | --- | --- | --- | --- |
|  | **ExtTa** | **DOX** | **ExtTa** | **DOX** |
| 0.05 | 2.085 | 1.64E-4 | 23.3384 | 0.25480 |
| 0.10 | 3.355 | 5.46E-4 | 16.4666 | 0.37234 |
| 0.15 | 4.503 | 0.00115 | 13.2686 | 0.47090 |
| 0.20 | 5.621 | 0.00202 | 11.2777 | 0.56197 |
| 0.25 | 6.750 | 0.00321 | 9.86060 | 0.65034 |
| 0.30 | 7.921 | 0.00481 | 8.76919 | 0.73883 |
| 0.35 | 9.159 | 0.00694 | 7.88295 | 0.82959 |
| 0.40 | 10.492 | 0.00980 | 7.13502 | 0.92459 |
| 0.45 | 11.954 | 0.01362 | 6.48457 | 1.02588 |
| 0.50 | 13.582 | 0.01882 | 5.90478 | 1.13589 |
| 0.55 | 15.432 | 0.02601 | 5.37682 | 1.25770 |
| 0.60 | 17.581 | 0.03617 | 4.88665 | 1.39549 |
| 0.65 | 20.141 | 0.05102 | 4.42301 | 1.55529 |
| 0.70 | 23.290 | 0.07370 | 3.97601 | 1.74636 |
| 0.75 | 27.330 | 0.11048 | 3.53593 | 1.98398 |
| 0.80 | 32.821 | 0.17560 | 3.09163 | 2.29594 |
| 0.85 | 40.966 | 0.30775 | 2.62774 | 2.73998 |
| 0.90 | 54.991 | 0.64840 | 2.11741 | 3.46528 |
| 0.95 | 88.477 | 2.16060 | 1.49395 | 5.06371 |
| 0.97 | 124.104 | 5.08757 | 1.16564 | 6.63253 |

**SUPPLEMENTARY TABLE 2 - Dose reduction index (DRI) for ExtTa and 5-FU for a given inhibition effect in HCT116 cell line**

*Values calculated at experimental points. DRI: dose reduction index, ExtTa: *T. asperelloides* ethanolic extract; DOX: doxorubicin; Fa: fractional inhibition.

| Fa | µg/mL | | DRI* | |
| --- | --- | --- | --- | --- |
|  | **ExtTa** | **5-FU** | **ExtTa** | **DOX** |
| 0.05 | 0.003 | 0.061 | 0.18530 | 2.26633 |
| 0.10 | 0.018 | 0.155 | 0.35959 | 2.01921 |
| 0.15 | 0.051 | 0.278 | 0.54210 | 1.87991 |
| 0.20 | 0.114 | 0.430 | 0.73841 | 1.78141 |
| 0.25 | 0.222 | 0.619 | 0.95314 | 1.70396 |
| 0.30 | 0.395 | 0.849 | 1.19125 | 1.63906 |
| 0.35 | 0.668 | 1.132 | 1.45869 | 1.58226 |
| 0.40 | 1.093 | 1.482 | 1.76305 | 1.53090 |
| 0.45 | 1.751 | 1.918 | 2.11437 | 1.48321 |
| 0.50 | 2.779 | 2.470 | 2.52645 | 1.43793 |
| 0.55 | 4.410 | 3.181 | 3.01884 | 1.39403 |
| 0.60 | 7.067 | 4.118 | 3.62041 | 1.35061 |
| 0.65 | 11.554 | 5.390 | 4.37582 | 1.30676 |
| 0.70 | 19.542 | 7.186 | 5.35820 | 1.26148 |
| 0.75 | 34.853 | 9.865 | 6.69674 | 1.21343 |
| 0.80 | 67.589 | 14.176 | 8.64416 | 1.16067 |
| 0.85 | 150.705 | 21.989 | 11.7745 | 1.09986 |
| 0.90 | 437.196 | 39.394 | 17.7507 | 1.02398 |
| 0.95 | 2,442.14 | 101.029 | 34.4475 | 0.91233 |
| 0.97 | 8,305.07 | 197.454 | 55.2120 | 0.84038 |
