# Supplementary figures and images for: Unveiling the anticancer potential of the ethanolic extract from Trichoderma asperelloides
Source: Front Pharmacol. 2024 May 1;15:1398135. doi: 10.3389/fphar.2024.1398135 (PMC11094271; doi:10.3389/fphar.2024.1398135)

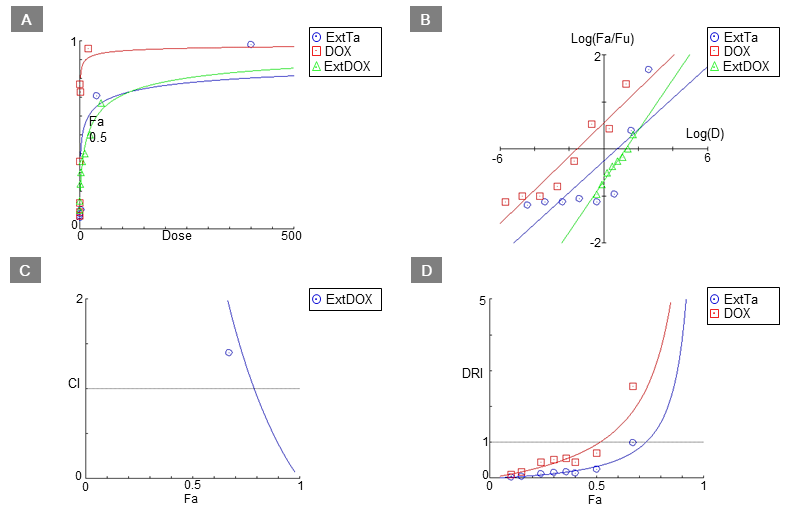

Supplement: Supplementary file 2 [file Image2.TIF]

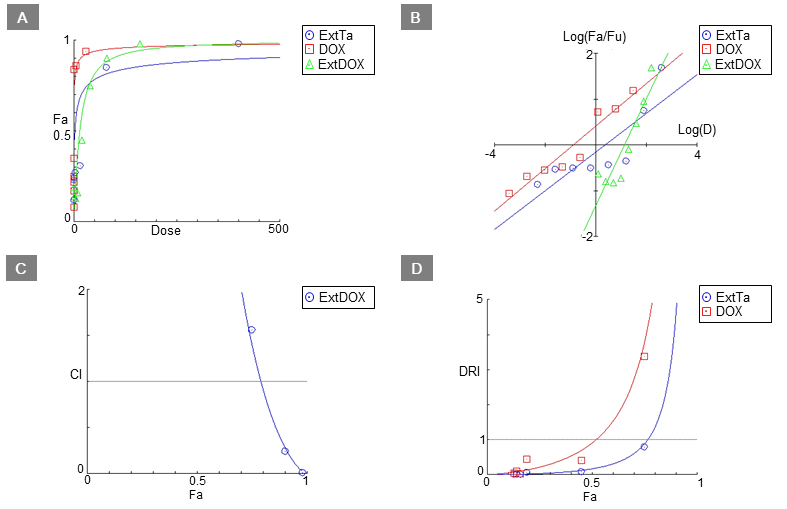

Supplement: Supplementary file 3 [file Image1.TIF]
